# THE ROLE OF TECHNOLOGY IN SUPPORTING ENGAGEMENT IN OLDER ADULTS: FINDINGS FROM THE PRISM 2.0 TRIAL

**DOI:** 10.1093/geroni/igae098.0039

**Published:** 2024-12-31

**Authors:** Sara Czaja, Walter Boot, Neil Charness, Wendy Rogers

**Affiliations:** Weill Cornell Medicine/Center on Aging and Behavioral Research, New York, New York, United States; Weill Cornell Medicine, New York, New York, United States; Florida State University, Tallahassee, Florida, United States; University of Illinois Urbana-Champaign, Champaign, Illinois, United States

## Abstract

Technology applications can play a key role in enhancing social and cognitive engagement, decreasing loneliness, and facilitating access to resources among older adults. This presentation will focus on data from the cross-site randomized field trial that evaluated the PRISM 2.0 system, an integrated software system (developed by the Center for Research and Education on Aging and Technology Enhancement), designed using a user-centered design approach, that fostered social and cognitive engagement and access to information and resources. PRISM 2.0 was compared to a Standard Tablet Control condition, where participants received a computer tablet without the PRISM 2.0 software and the same amount of training and contact as those in the PRISM 2.0 condition. The trial was conducted in diverse living contexts including rural locations, senior housing, and assisted living communities (ALC). The sample included 245 adults aged 64-99 yrs. that were randomized within living context to either the PRISM 2.0 or the Standard Tablet Control condition. Data were collected at baseline, and 6-and 9-months post randomization. Findings will be presented on the impact of access to the PRISM 2.0 software system on social isolation, loneliness, social support, quality of life, and technology proficiency. Information will also be presented regarding the unique challenges of implementing PRISM 2.0 in the various contexts. Overall, the findings provide strong evidence that access to technology is beneficial to aging adults and underscore the importance of adapting technology to the needs and preferences of the user and providing adequate instructional support.

